# DoliClock: a lipid-based aging clock reveals accelerated aging in neurological disorders

**DOI:** 10.18632/aging.206266

**Published:** 2025-06-04

**Authors:** Djakim Latumalea, Maximilian Unfried, Diogo Barardo, Jan Gruber, Brian K. Kennedy

**Affiliations:** 1 Healthy Longevity Translational Research Programme, Yong Loo Lin School of Medicine, National University of Singapore, Singapore; 2 Institute for Life Sciences and Technology, Hanze University of Applied Sciences, Groningen, The Netherlands; 3 Department of Biochemistry, Yong Loo Lin School of Medicine, National University of Singapore, Singapore; 4 Department of Physiology, Yong Loo Lin School of Medicine, National University of Singapore, Singapore; 5 Centre for Healthy Longevity, National University of Singapore, Singapore; 6 Science Division, Yale-NUS College, Singapore

**Keywords:** aging clock, down syndrome, autism, schizophrenia, dolichol

## Abstract

Aging is a multifaceted process influenced by intrinsic and extrinsic factors, with lipid alterations playing a critical role in brain aging and neurological disorders. This study introduces DoliClock, a lipid-based biological aging clock designed to predict the age of the prefrontal cortex using post-mortem lipidomic data. Significant age acceleration was observed in autism, schizophrenia, and Down syndrome. Additionally, an increase in entropy around age 40 suggests dysregulation of the mevalonate pathway and dolichol accumulation. Dolichol, a lipid integral to N-glycosylation and intracellular transport, emerged as a potential aging biomarker, with specific variants such as dolichol-19 and dolichol-20 showing unique age-related associations. These findings suggest that lipidomics can provide valuable insights into the molecular mechanisms of brain aging and neurological disorders. By linking dolichol levels and entropy changes to accelerated aging, this study highlights the potential of lipid-based biomarkers for understanding and predicting biological age, especially in conditions associated with premature aging.

## INTRODUCTION

Neurological syndromes including Parkinson’s and Alzheimer’s disproportionately affect the older population [1]. Moreover, these disorders (and others) are major contributors to mortality [2] and cause a significant financial burden [3]. The primary risk factor for developing most neurological disorders is aging [4]. While aging is characterized by a loss of physiological function and an exponential increase in mortality [5], aging is also a highly heterogeneous process [6] influenced by a range of intrinsic and extrinsic factors [7].

Lopez-Otin et al. [8] proposed a set of hallmarks of aging, which can be further classified into three distinct categories: primary, antagonistic, and integrative hallmarks. These hallmarks aim to capture the underlying causes of damage, the key responses to such damage, and the resulting effects responsible for the ultimate functional decline of aging organisms.

Alterations in lipid composition play a critical role as they are involved in metabolic energy, homeostasis, and cell signaling [9, 10]. In addition, aging is known to significantly alter the brain lipidome [11], which will impact the hallmarks of aging, such as proteostasis [12], and result in decreased resilience and neuronal plasticity [13]. It therefore stands to reason that age-dependent alterations in the lipidome may contribute to brain aging and increase the risk of developing neurological disorders [4]. Evidence already exists that lipid alterations contribute to specific age-dependent neurological disorders. For instance, dysregulated lipid homeostasis has been linked to inflammaging which plays a role in the etiology of Alzheimer’s disease [14]. In addition, lipid peroxidation is positively correlated with Alzheimer’s disease, Down syndrome, and other neurological disorders [15]. Although neurological disorders are mostly found in aged individuals, they can develop at a younger age under certain contexts. Prenatal stress and other environmental stressors can increase the risk of developing neurological disorders, such as schizophrenia, and autism spectrum disorders [16–18]. People with Down syndrome, schizophrenia, and autism also tend to die at a younger age [19–21], which may indicate accelerated aging.

Age acceleration can be measured using aging clocks, which may provide insight into the underlying factors that contribute to the aging process. Numerous aging clocks have been developed to predict biological age. The first prominent clock, developed by Hannum et al. [22], was based on DNA methylation data from whole blood. Subsequently, various other aging clocks using DNA methylation data have been developed and proposed [23, 24]. This has been followed by the development of aging clocks utilizing other omics data, such as transcriptomics [25], proteomics [26], lipidomics [27], or a combination of various data sources [28, 29]. Most of these models are considered first-generation aging clocks as they predict chronological age. However, chronological age may not always be the most accurate marker, which has prompted researchers to directly predict mortality [30, 31]. These clocks are known as second-generation clocks.

Horvath et al. [32] also used their aging clock [23] on brain tissue from individuals with Down syndrome showing a significant age acceleration effect. In addition, Cole et al. [33] utilized neuroimaging data to identify factors associated with age acceleration in individuals with Down Syndrome. Both studies found significant age acceleration and showed that age acceleration is measurable through DNA methylation and neuroimaging.

Age acceleration has also been proposed in schizophrenia, although studies are conflicting [34]. Higgins Chen et al. [35] explored 14 epigenetic clocks and found that 3 mortality-based clocks were able to identify significant acceleration in schizophrenia patients. Conversely, other studies utilizing epigenetic clocks based on chronological age or mortality data did not observe significantly accelerated tissue specific brain aging [36, 37]. This may be caused by the relatively low number of samples. It seems that DNA methylation clocks lack the capability to measure aging in individuals with schizophrenia, whereas mortality clocks are proficient, although they depend on clinical parameters. Developing an aging clock based on lipids provides another perspective.

Additionally, there is very limited data on brain aging in individuals with Autism [22, 38], highlighting a critical gap in our understanding of how Autism may influence the aging process.

In this study, we aimed to determine if lipidomics data from the prefrontal cortex could predict biological age. While several clocks have been devised for human brain tissue [32, 39, 40], they predominantly rely on DNA methylation data, or on transcriptomic data [41, 42]. However, to our knowledge, no model based on lipids from the prefrontal cortex has been developed thus far, making this study a novel exploration in the field of aging clocks. Lipids are integral to understanding the relationship between aging and neurological disorders, given that they constitute approximately 40% of the dry-weight gray matter [43], with the brain exhibiting the highest diversity of lipid species [44]. These findings suggest that a lipid-based clock might pinpoint aging-associated lipids, potentially shedding light on conditions like Down syndrome, schizophrenia, and autism. Additionally, we identify molecules strongly linked with age and propose them as predictive biomarkers [45, 46]. Our study demonstrates that variations in brain lipids suffice for estimating biological age.

## RESULTS

In the present study, we utilized the publicly available dataset by Yu et al. [11]. We retained 242 samples and 163 lipid species, selected for unique chemical formulas to ensure dataset granularity. Among 39,446 lipid concentration values, 1.98% were missing, with 47% of lipids having at least one missing value. The dataset included 195 samples without neurological disorder (WND), 27 samples with schizophrenia (SZ), 15 samples with autism spectrum disorder, and 5 samples with Down syndrome (DS). Supplementary Table 1 provides a comprehensive breakdown of the preprocessed dataset. No outliers were identified. This preprocessing step ensured that the dataset was suitable for downstream modeling and analysis by reducing noise and maintaining data integrity.

### Identifying a robust model

To develop a predictive model based on lipidomic profiles, we first evaluated multiple machine learning approaches to determine the most reliable method for age prediction. Twenty-six machine learning models were trained using 100 bootstrap iterations to identify the most generalizable model. Linear regression models demonstrated the best performance (Supplementary Figure 1), and detailed model performance metrics are presented in Supplementary Table 2. Based on these findings, we developed an Elastic Net model and applied principal component analysis (PCA) to reduce dimensionality and mitigate noise. PCA projections were used as input for the model, a strategy commonly employed in similar studies [27, 39, 47]. To ensure robustness, we bootstrapped the data 10,000 times with replacement, stratifying by age group, sex, and ethnicity, and trained Elastic Net models using WND samples. During training, we included ethnicity, sex, and the post-mortem interval (PMI) as covariates to account for additional variance. This approach enables us to capture lipidomic patterns while minimizing overfitting and enhancing model interpretability.

### Exploring principal components and lipid patterns

After establishing a robust predictive model, we proceeded to investigate how lipidomic features, particularly the principal components, correlated with age and other biological variables. To investigate whether there was a relationship between the principal components and the metadata, we conducted a correlation analysis (Supplementary Figure 2). The first principal component showed no substantial correlation with the meta data, but was enriched for PG(0-20:0/22:4), a glycerophospholipid involved in membrane signaling, likely due to its high variance. In contrast, principal components two and three exhibited significant Pearson correlation coefficients (r) with Shannon entropy (r = −0.30, *P* < 0.001 and r = 0.30, *P* < 0.001, respectively). Dolichol-19 and dolichol-20 were identified as primary contributors to entropy in 31% of samples. When entropy was recalculated using only dolichols, a striking correlation with chronological age (r = 0.92, *P* < 0.001) emerged. These findings suggested that dolichols could serve as potential age-associated markers, warranting further investigation into their role in aging processes.

### Entropy and aging

**Figure 1:**
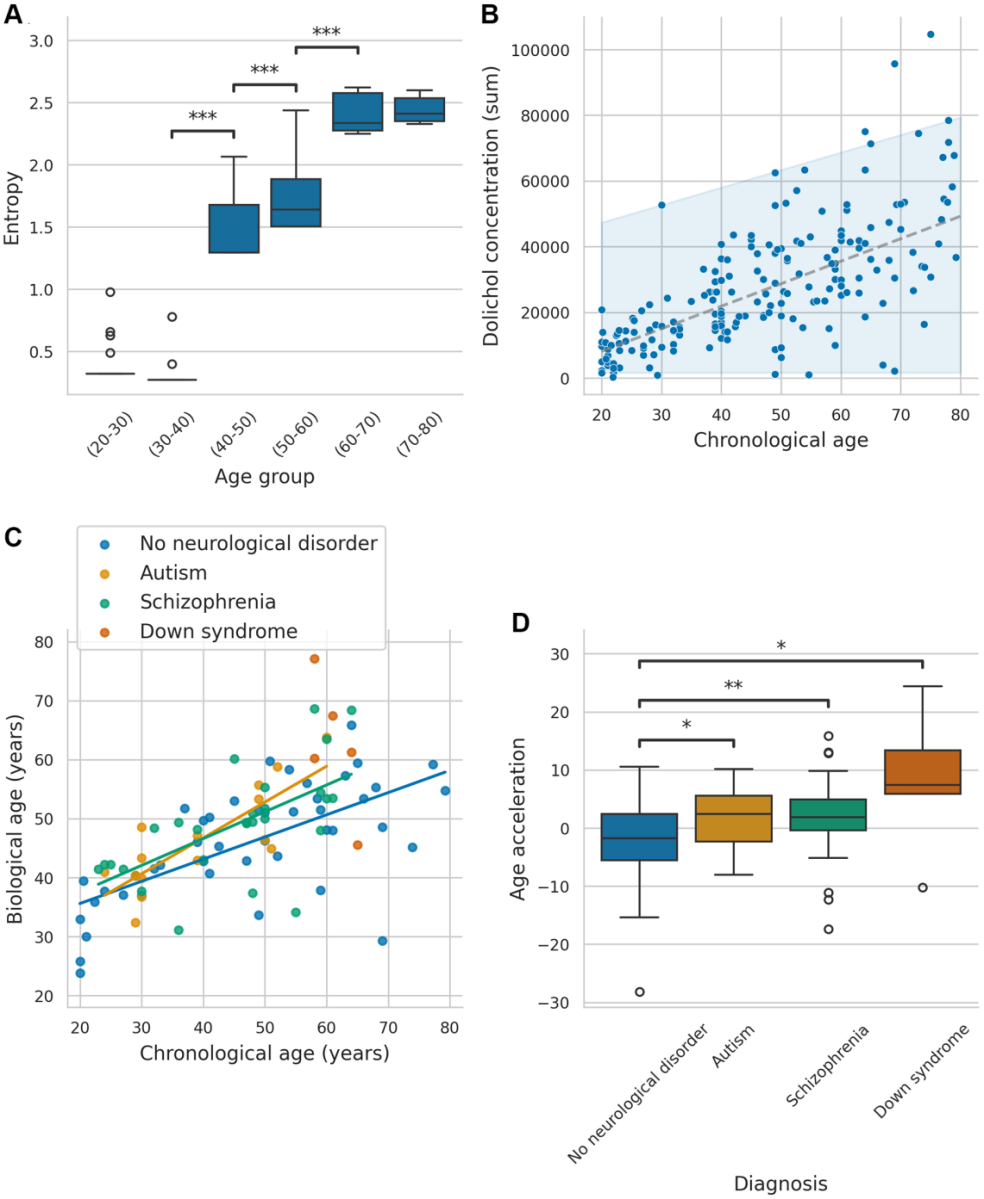
**Association between entropy, biological age, and biomarkers across neurological conditions.** (**A**) Boxplots of entropy across different age groups (only significant *P*-values are annotated: *P* < 0.05 = ^*^, *P* < 0.01 = ^**^, *P* < 0.001 = ^***^). (**B**) Behavior of summed dolichol concentration through age, with a 95% confidence interval. (**C**) Predicted values of the models for all samples, including groups with no neurological disorder, autism, schizophrenia, and Down syndrome. (**D**) Boxplot comparing age acceleration across the different groups (Only significant *P*-values are annotated: *P* < 0.05 = ^*^, *P* < 0.01 = ^**^, *P* < 0.001 = ^***^).

Given the strong correlation between entropy and age, we further examined how entropy levels varied across different age groups. To explore the relationship between entropy and aging, samples were divided into six age bins (20–80 years, 10-year interval), and entropy levels were compared using the Mann-Whitney *U*-test. Samples aged 40–50, 50–60, and 60–70 exhibited significantly higher entropy levels than their younger counterparts (*P* < 0.001 for each group; significant after Holm-Bonferroni correction). These results indicate a substantial increase in entropy around the age of 40–50 (Figure 1A). Interestingly, no significant differences in entropy were observed between ASD, SZ, and DS samples, and corresponding controls (*P* > 0.05 after Holm-Bonferroni correction; Supplementary Figure 3), suggesting that age-related changes in entropy were more pronounced than disorder-specific patterns. To further characterize lipidomic aging patterns, we examined the specific principal components that contributed most significantly to age-related variation.

### Aging-associated principal components

Principal component five emerged as a key aging-related feature, exhibiting a strong correlation with age (r = 0.61, *P* < 0.001) and receiving the highest coefficient in the Elastic Net model. The loadings of principal component five were dominated by dolichol-19, dolichol-20, and specific glycerophospholipids such as PG(17:1(9Z)/0:0) and PG(22:6). These findings indicated that principal component five encapsulates a broader set of aging-related processes beyond dolichol metabolism.

Given that ethnicity and sex can influence lipidomic profiles, we next examined whether lipid composition varied across different populations.

### Ethnicity, sex and lipid profiles

Ethnicity emerged as a significant factor influencing lipid composition, with principal component six positively correlated with Han Chinese ethnicity (r = 0.54, *P* < 0.001) and negatively correlated with Caucasian ethnicity (r = −0.32, *P* < 0.001). These findings suggest distinct lipid concentration patterns across ethnic groups, emphasizing the need to account for population-specific lipid profiles in lipidomic aging models. In contrast, surprisingly, sex exhibited no significant correlation with any principal component, indicating that sex-based differences play a limited role in lipid variance within this dataset. Given the observed impact of ethnicity on lipid composition, we next explored how lipid levels change with chronological age to assess whether certain lipidomic shifts are consistent aging markers across populations.

### Lipid trends with age

To identify molecules significantly associated with age, we conducted pairwise comparison between different age groups using the Mann-Whitney *U*-test (Supplementary Table 3). Multiple lipids exhibited significant increases with age after Bonferroni correction, particularly between the 0–20 and 20–40 age groups. Notably, dolichols showed a strong linear increase with age and increasing variance (Figure 1B). Specifically, dolichol-19 C95H160NO and C95H157O, as well as dolichol-20 C100H164ONa, C100H165O, C100H168NO, demonstrated significant increases between age groups 0–20, and 20–40 (*P* < 0.00007 for each), and between groups 20–40 and 40–60 (*P* < 0.00007 for each). However, only dolichol-20 C100H164ONa exhibited a significant increase between groups 40–60 and 60–80 (*P* < 0.00007), with no significant increases observed between groups 60–80 and 80–100, possibly due to limited sample size and high variance. The log fold changes for these comparisons are shown in Supplementary Table 4.

Based on these findings, it was hypothesized that dolichol may serve as a biomarker for predicting biological age, given its significant and consistent increase across age groups. These results reinforce the role of dolichols as aging markers, with their accumulation reflecting progressive biological aging.

### DoliClock

To test whether these findings could be leveraged for biological age prediction, we developed a lipid-based aging clock, DoliClock, an Elastic Net model trained exclusively on dolichol lipids. The model incorporated sex, ethnicity, and PMI as covariates to account for additional sources of variation. Using the default Elastic Net parameters, we performed 10,000 bootstrap iterations stratified by ethnicity and sex. The model achieved a median absolute error of 8.96 years, with the median-performing model selected for further analyses. The distribution of the performance can be found in Supplementary Figure 4. These results demonstrate that a lipid-based clock can reliably estimate biological age, reinforcing the role of dolichols in aging prediction.

To further assess the applicability of DoliClock, we tested its performance in samples with neurological disorders to determine whether the pace of aging differs in these conditions. DoliClock was applied to predict the biological ages ASD and SZ samples, as shown in Figure 1C. We assessed whether the relationship between chronological age and DoliClock-predicted age differed across groups using regression analysis, excluding DS samples due to their limited number in comparable age ranges. Chronological age was a significant predictor of DoliClock in all groups (*P* < 0.001), with an estimated increase of 0.38 units per year in controls. Although ASD samples showed a trend toward a steeper aging slope (an additional 0.23 units/year), the slope was not significantly greater (*P* = 0.10). Similarly, the slope for SZ samples (an additional 0.08 units/year) was not significantly greater in comparison with samples without neurological disorders (*P* = 0.27).

To further evaluate aging dynamics, we calculated age acceleration (Figure 1D). ASD, SZ, and DS samples exhibited significantly greater age acceleration compared to WND samples (*P* = 0.047, *P* = 0.008, and *P*=0.015, respectively; all significant after Holm-Bonferroni correction), suggesting these conditions are associated with accelerated aging. This highlights the potential of dolichol-based clocks for identifying biological age deviations in neurological disorders.

Next, we examined which lipid species contributed most to DoliClock’s predictions, providing insight into the molecular basis of its accuracy.

**Figure 2:**
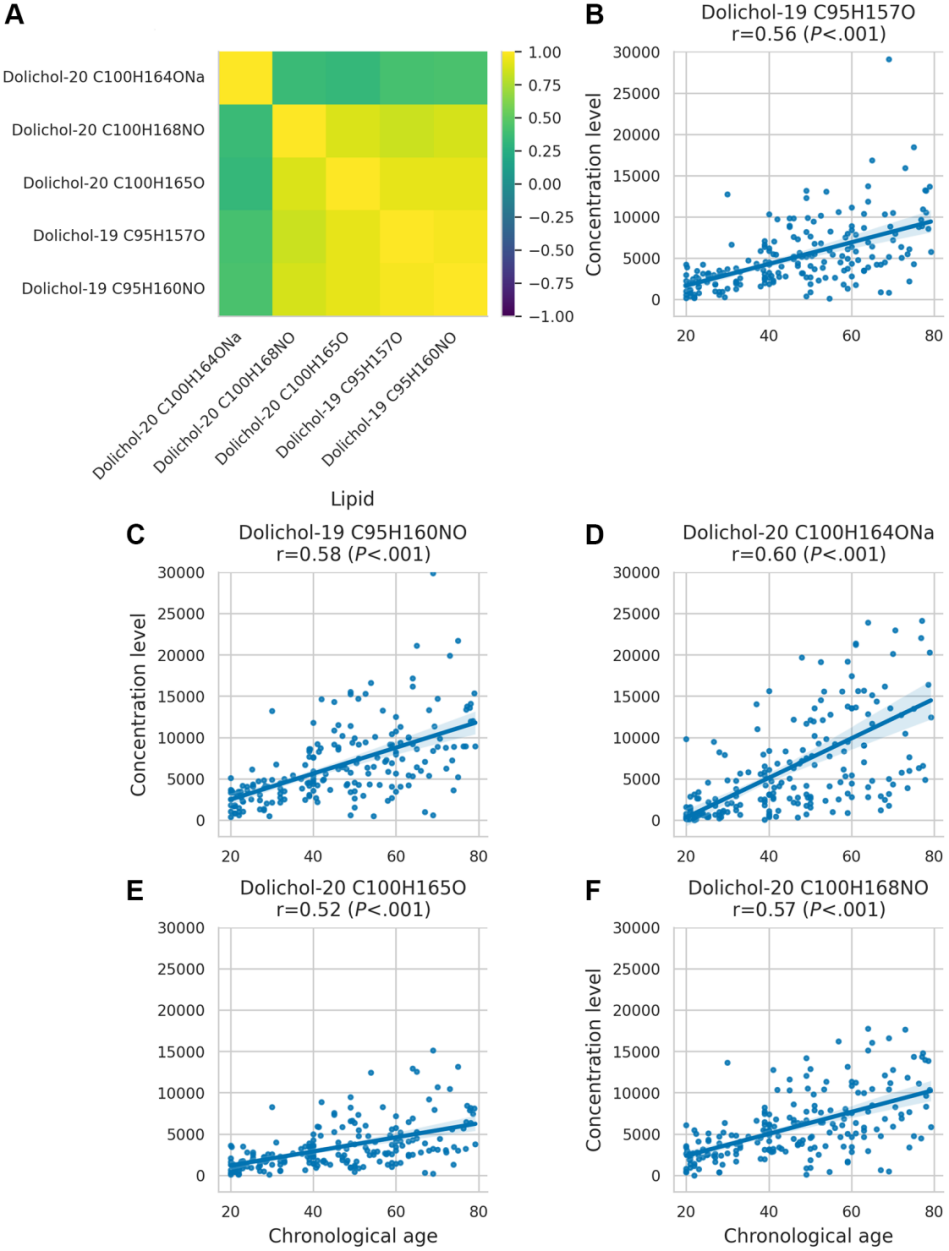
**Dolichol correlation matrix and age-related concentration changes.** (**A**) Correlation matrix illustrating the relationships between different dolichol subspecies. (**B**–**F**) Scatter plots showing the behaviour of dolichol subspecies across age.

A correlation analysis of dolichol species (Figure 2A) revealed strong correlations among most dolichols, except C100H164ONa, which exhibited a distinct pattern. Unlike other dolichols, dolichol-20 C100H164ONa showed weaker correlations with related species, suggesting a unique regulatory mechanism or functional divergence in its role in aging.

To better understand how dolichol levels change over time, we analyzed their variability across the lifespan. This analysis revealed that the variance of dolichol levels increases with age (Figure 2B–2F), with a pronounced rise observed around the age of 40 (*P* < 0.001 for all dolichols, Levene’s test; all significant after Holm-Bonferroni correction). This increasing heterogeneity suggests that age-related changes in dolichol concentrations reflect not only chronological aging but also growing individual differences in biological aging trajectories.

**Figure 3:**
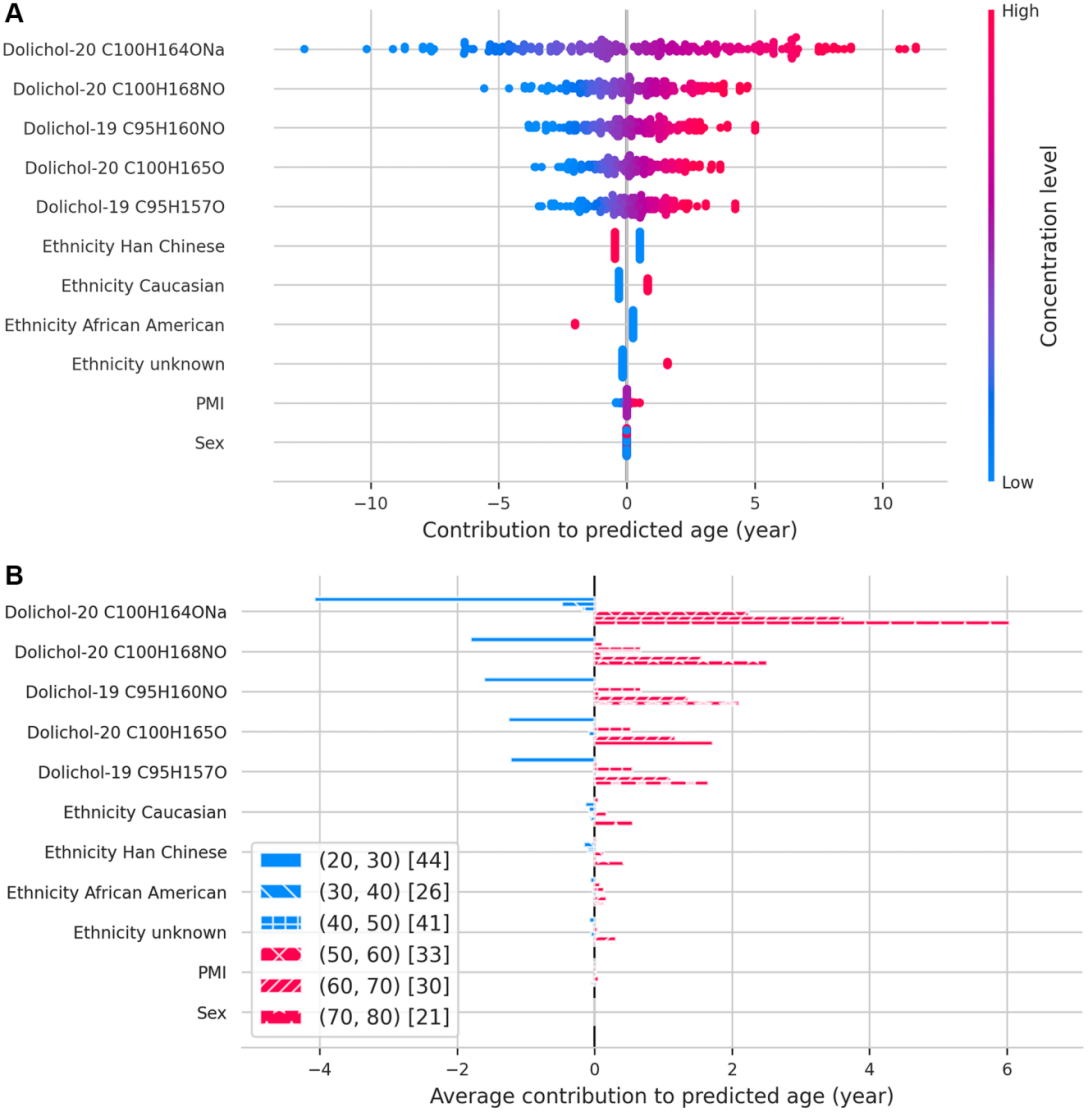
**SHAP values illustrating the contribution of each feature to predicted age.** (**A**) Beeswarm plot displaying the relative importance and direction of influence of each feature on age predictions. (**B**) Bar plot showing how each feature contributes to age predictions across different age groups, binned by decade.

Building on these findings, we assessed the predictive value of individual dolichols for biological aging. Feature importance analysis using SHAP values identified dolichol-20 C100H164ONa as the most influential predictor of biological age (Figure 3A). Lower concentrations of dolichol-20 and dolichol-19 were linked to younger predicted ages, while moderate or high concentrations corresponded to older predicted ages. This relationship between dolichol and chronological age changes over time, as can be seen across the different age groups (Figure 3B); in younger individuals, lower levels correlate with a more youthful biological profile, whereas in older individuals, rising dolichol levels signal biological aging due to their progressive accumulation with age. These results further support the role of dolichols as robust lipid-based biomarkers of aging.

Beyond individual lipid markers, we also investigated the influence of ethnicity on DoliClock predictions, as population differences in lipid metabolism could impact biological age estimates.

Ethnicity had a minor but measurable effect on DoliClock predictions. Han Chinese samples were generally predicted to be younger, whereas Caucasian samples were predicted to be older. However, differences in age distributions across ethnic groups in the training data likely contributed to these patterns (Supplementary Figure 5). This underscores the importance of considering demographic factors when applying lipid-based aging models across populations.

## DISCUSSION

### Age acceleration

Our study identified significant age acceleration in samples with autism, schizophrenia, and Down syndrome. The findings for schizophrenia align with and extend conflicting outcomes reported in prior studies using epigenetic clocks, which assess chronological age and mortality [35–37]. This suggests that lipid-based aging clocks may provide additional insights where epigenetic clocks have shown inconsistencies.

Lipidomic profiling, as applied in this study, appears to capture distinct age-related signatures in schizophrenia, offering an alternative perspective on aging processes in this population. Similarly, while further exploration into brain aging in autism remains necessary [20, 38], our results demonstrate significant age acceleration in individuals with autism. This aligns with prior research suggesting a potential role of genetic factors linked to both aging and autistic traits or the indirect influence of these traits on lifestyle, thereby modulating the aging process [48]. For samples with Down syndrome, our findings corroborate earlier studies that documented premature aging through epigenetic markers and neuroimaging [32, 33]. This consistency highlights the utility of lipidomic analysis in understanding age-related changes in Down syndrome.

### Entropy and age-related dysregulation

Furthermore, our study highlights a striking escalation in entropy, particularly around the age of 40. This suggests a possible dysregulation in the mevalonate pathway, potentially leading to the accumulation of dolichol. The observed strong correlation between entropy and chronological age supports the hypothesis that entropy reflects age-dependent dysregulation in dolichol-related pathways.

Dolichols, a subclass of polyprenols, are essential for intracellular transport [49], N-glycosylation [50], and play a pivotal role in the mevalonate pathway [51], which synthesizes key molecules such as cholesterol, dolichol, and ubiquinone. Alterations in the concentration levels of these molecules, particularly the age-related increase in dolichol [52–56], suggest a dysregulation of the mevalonate pathway. This dysregulation may be driven by an age-related increase in HMG-CoA reductase, the rate-limiting enzyme of this pathway [57]. Since these pathways are integral to cellular function, disruptions may have significant consequences for age-related decline in brain function.

Our findings align with those of Shen et al. [58], who observed a transition in lipid metabolism around age 40, indicating a fundamental shift in systemic lipid regulation. The authors identified a module related to plasma lipoprotein remodeling, a process that relies on the glycosylation of lipoproteins and their receptors, which in turn depends on dolichol biosynthesis. If dolichol metabolism is altered, glycosylation efficiency may decline, potentially impacting lipoprotein metabolism and contributing to the lipid remodeling changes observed at midlife. These findings suggest that the entropy increase we report may reflect a broader systemic reorganization of lipid homeostasis during aging.

Recent research further supports the role of entropy and stochastic variation in aging. Studies on aging clocks emphasize that increasing biological disorder is a fundamental feature of aging, with stochastic fluctuations alone being sufficient for predicting biological age [59, 60]. Moreover, insights into epigenetic aging suggest that while some changes occur randomly, others are biologically regulated [61]. This dual framework may apply to lipidomic entropy as well, where dolichol accumulation could result from both passive molecular drift and active metabolic shifts. The observed alterations reinforce the link between entropy and lipidomic dysregulation. Collectively, these findings highlight entropy as a key characteristic of biological aging and support the relevance of lipidomic biomarkers in aging research.

### Dolichol as an aging biomarker

Dolichol’s potential as an aging biomarker is supported by extensive research across various organisms, including mice [62–64], rats [52, 65–70], drosophila [71], and humans [53–55, 72–75]. Dolichol, a compound comprising a variable number of isoprene units depending on the species [76], has garnered attention for its association with aging processes [77, 78]. Studies have reported an accumulation of dolichol in multiple tissues with age, although concentrations may vary among tissue types. For instance, research on rats’ liver revealed that dolichol concentration increases in tandem with HMG-CoA reductase levels [66], with caloric restriction shown to retard dolichol accumulation [67, 68]. Similarly, investigations on brain tissue have shown a several-fold increase in dolichol concentration during aging and development, suggesting potential developmental roles [52]. Studies in humans have echoed these findings, with dolichol levels increasing dramatically in adults compared to neonates [72]. Notably, specific dolichol molecules, such as those with isoprene unit 17 up to 21, have been found to be particularly abundant in brain tissue [73]. However, studies have also linked elevated dolichol levels to neurodegenerative diseases, suggesting a potential link to lysosomal dysfunction [53]. Additionally, individuals with conditions like Down syndrome and autism have been observed to exhibit elevated dolichol levels [54, 55], further supporting its role as a biomarker of both aging and neurological conditions.

Dolichol also plays critical roles in organelle transport and N-glycosylation [49, 50], serving as the mevalonate pathway's end product [51]. Its role as a lipid carrier for glycan precursors suggests a potential link between glycan and dolichol biomarkers, hinting at a mechanistic relationship [79, 80]. Additionally, caloric restriction has shown promise in reducing dolichol accumulation, although its effectiveness may vary depending on the duration and timing [66, 67].

Emerging evidence also suggests dolichol may protect aging membranes against free radical damage, potentially slowing biological aging [68]. Conversely, an unstable dolichol system could accelerate aging processes [81]. Another hypothesis posits that dolichol accumulation might involve low-density lipoprotein dysregulation, impairing lysosomal function leading to autophagic degradation [68, 82].

### Tissue-specific versus systemic aging

While our study utilized brain tissue, the regression on chronological age raises questions about whether observed changes reflect tissue-specific aging or systemic aging processes. Brain tissue might uniquely capture certain age-related signatures, but further research is needed to determine whether similar patterns occur in other tissues. Distinguishing between tissue-specific and systemic effects is crucial for understanding the role of dolichol in aging. Understanding whether lipidomic aging patterns are exclusive to the brain or extend to peripheral tissues could help determine the broader applicability of DoliClock and similar models.

### Limitations and confounding variables

The post-mortem nature of our dataset necessitates consideration of confounding factors. Cause of death, medication usage, and post-mortem interval (PMI) could all influence lipid levels. Notably, the disproportionately high number of samples with a PMI of exactly 14 among healthy individuals suggests this value might be a default placeholder rather than an exact measurement. This could introduce bias in our analysis, though the extent of its impact remains unclear. Additionally, the unknown medication status of individuals with schizophrenia, a condition often treated with lipid-altering drugs, could further confound results [83]. This means we were unable to include any medication information as a covariate or assess its potential influence on lipidomic profiles. Given that antipsychotic and other neuropsychiatric medications are known to alter lipid metabolism, it is possible that some of the variance observed in our dataset is attributable to medication effects rather than underlying biological aging processes. However, without access to medication records, we are unable to disentangle these effects from disorder-related lipidomic changes. Furthermore, ethnicity also represents a potential confounding variable, as genetic, environmental, and lifestyle differences across ethnic groups may influence lipid metabolism and other biological aging markers. While our models account for key covariates, unmeasured ethnic-specific effects could contribute to variability in the results. Addressing these factors in future research will be critical for refining dolichol's utility as an aging biomarker and improving their robustness across diverse populations.

Another major limitation of our study is the limited generalizability of findings due to sample size constraints. Biological variability, including differences in genetic background, health conditions, and lifestyle factors, may not be fully captured within our cohort, potentially reducing the broader applicability of our results. This issue is particularly pronounced in our analysis of samples with Down syndrome, where we observe significant age acceleration. Given that this finding is based on only five samples, it remains unclear whether the observed lipidomic changes represent a true biological signal or random variation. Larger sample sizes will be necessary to confirm whether these alterations are consistent features of DS aging.

### Future directions

Future research should prioritize validating the findings of this study through external datasets or experimental approaches, given the inherent limitations of post-mortem tissues. Understanding the mechanisms linking dolichol and aging, particularly the functional distinctions between dolichol-19 and dolichol-20, is crucial for unraveling their roles in the aging process. Investigating their potential contributions to autophagic degradation and lipid peroxidation may also clarify their involvement in cellular aging.

Additionally, a key avenue for exploration is the comprehensive analysis of dolichol levels across various tissues to uncover clinical aging markers or surrogate indicators for brain aging. Since dolichol concentrations vary significantly between tissue types, integrating lipidomic data from multiple sources could enhance the precision and generalizability of age-related biomarkers. Notably, since DoliClock was developed using prefrontal cortex lipidomic data, its immediate clinical applications are constrained to post-mortem analysis. However, one promising direction for translation is investigating whether the lipid aging signatures identified in brain tissue are reflected in biofluids, such as blood plasma or cerebrospinal fluid. If similar lipid patterns can be detected in accessible samples, DoliClock could serve as a foundation for developing non-invasive biomarkers of brain aging and neurological disorders, facilitating earlier diagnosis and monitoring of neurodegenerative conditions.

Beyond diagnostics, DoliClock could also play a role in therapeutic intervention planning. By providing insights into how lipid metabolism contributes to accelerated aging in neurological disorders, it could be leveraged as a research tool to assess whether interventions targeting lipid metabolism—such as dietary modifications, statins, or metabolic regulators—influence lipid aging signatures in experimental models or patient cohorts. Although direct therapeutic applications remain speculative, such investigations could pave the way for novel strategies aimed at modulating lipid homeostasis to mitigate age-related dysfunction.

Furthermore, the interplay between entropy, dolichol, and aging presents an exciting frontier. The observed entropy increase and dolichol-19 concentration shift around age 40 suggest that combining insights from information theory and lipidomics could yield novel perspectives. This interdisciplinary approach may uncover unique molecular signatures of aging, offering deeper insights into its underlying biological mechanisms.

## CONCLUSION

This study explored the potential of lipidomic data to predict biological age in the prefrontal cortex and to identify critical lipid molecules linked to aging. We introduced DoliClock, a novel tool leveraging prefrontal cortex lipid profiles to estimate age. Notably, DoliClock uncovered previously unreported isotopic and chemical variations in dolichol molecules associated with age, highlighting its capacity to detect subtle, age-related biological effects.

DoliClock demonstrated robust performance in estimating biological age across a spectrum of individuals, including those with neurological disorders like Down syndrome, schizophrenia, and autism, which are commonly associated with accelerated aging phenotypes. Our findings revealed significant age acceleration in autism, schizophrenia, and Down syndrome.

Furthermore, we identified a notable increase in entropy around age 40. These results emphasize the potential of lipidomic data to inform biological age prediction and underscore its utility in advancing our understanding of lipid-based biomarkers of aging.

## MATERIALS AND METHODS

### Samples

This study utilized the publicly available dataset by Yu et al. [11], comprising 452 samples from various brain banks: NICHD Brain and Tissue Bank, Maryland Psychiatric Research Center, Maryland Brain Collection Center, Netherlands Brain Bank, Chinese Brain Bank Center, Harvard Brain Tissue Resource Center, and Autism Tissue Program. Samples, primarily gray matter from the anterior prefrontal cortex, weighed approximately 12.55 mg (±1.65). The dataset included 403 samples without neurological disorders (WND), 5 with Down syndrome (DS), 17 with autism spectrum disorder (ASD), and 27 with schizophrenia (SZ). Age ranges varied: WND samples from 0 to 99 years (median age 24 years, excluding 12 prenatal samples), DS samples from 58 to 65 years (median age 61 years), ASD samples from 18 to 60 years (median age 30 years), and SZ samples from 23 to 64 years (median age 48 years). Post-mortem intervals (PMI) ranged from 0 to 44 hours, with median values of 14, 6.2, 20.3, and 19.5 hours for WND, DS, ASD, and SZ samples, respectively. Ethnicity data were incomplete, labeled as unknown, thus limiting its use for matching case and control groups. Detailed sample statistics are provided in Supplementary Table 5. A comprehensive description of these data, the methodology, and availability can be found in the publication of Yu et al. in 2018 [11].

### Data analysis

#### Data preprocessing

To standardize ages, we adjusted reported values by subtracting a gestational period of 0.767 years. Subjects younger than 20 clustered in feature space (Supplementary Figure 6), likely reflecting ongoing brain development, while those over 80 were mostly Caucasian (Supplementary Figure 5). To address these biases, we limited our analysis to individuals aged 20 to 80. After filtering, the dataset contained 5,024 lipid species, of which 2,222 were annotated with LIPID MAP IDs (LM IDs). To manage degenerate LM IDs, we computed a conformity index, resulting in 360 unique lipid species mapped to LM IDs, molecular weights, and retention times. Isotopic variations were reviewed for discrepancies in m/z values, and mass searches against LIPID MAPS adduct lists were conducted across lipid classes in both ionization modes. Any unmatched values were treated as missing, resulting in a final dataset with 242 samples and 163 features.

Outliers were identified by running PCA and calculating the pairwise Euclidean distances between samples. The distances were then averaged per sample. An outlier was defined as any distance below the first percentile − 3 × the interquartile range or above the third percentile + 3 × the interquartile range.

#### Model development and bootstrapping

We trained and evaluated all models using bootstrap resampling to enhance robustness. A total of 100 bootstrap iterations were performed to select the best-performing null models, as additional iterations did not improve model selection. As some models are capable of capturing complex, nonlinear patterns, we did not use PCA at this stage. For both Elastic PCA and DoliClock, 10,000 bootstrap iterations were conducted to ensure stable estimates of biological age. Bootstrapping was performed with replacement, preserving the original sample size in each iteration. The resampling was stratified by age (5-year bins), sex, and ethnicity, ensuring demographic diversity in each bootstrap sample. Sex, ethnicity, and PMI were included as covariates to minimize potential confounding effects. Model performance was evaluated using out-of-bootstrap samples, which served as independent test sets, ensuring an unbiased performance assessment. All preprocessing steps were applied exclusively to the training data and projected onto the test set. Missing values were imputed using K-nearest neighbors (k = 5), followed by scaling and normalization via the Yeo-Johnson transformation [84]. Elastic PCA incorporated an additional PCA step before model training.

Statistical analyses were conducted using the Mann-Whitney *U*-test to compare entropy and lipid levels across age groups, as well as to assess age acceleration between diagnostic groups. Regression analysis was employed to compare the slopes of aging trends between groups, and Levene’s test was used to evaluate differences in lipid variance across age groups. For all hypothesis-driven comparisons, we applied the Holm-Bonferroni correction to control for multiple testing. Bonferroni correction (*P* < 0.00007) was used specifically for exploratory correlation analyses, given the higher number of comparisons.

Age acceleration was computed by fitting a linear regression model to the predicted and actual ages, with residuals representing deviations from the expected aging trajectory.

#### Dimensionality reduction, mutual information, and interpretation

For dimensionality reduction, we applied PCA, using singular value decomposition (SVD) to decompose the data’s correlation matrix into eigenvalues and eigenvectors, represented as *S* = *QΛQ^T^* [85]. PCA was implemented via the scikit-learn library [86] for visualization and subsequent analysis. To further explore relationships among features, eigenvectors were rescaled based on mutual information [87], calculated following the method of Platt et al. [88].

Elastic net was employed to identify sparse, informative features while minimizing overfitting [89]. Using the ElasticNet class from scikit-learn [86], PCA loadings were multiplied by elastic net coefficients to determine feature importance for models using PCA. Feature importance for models utilizing raw data we assessed using model coefficient and SHAP values [90].

#### Entropy

Shannon entropy [91], was calculated to assess information content, under the assumption of stable prefrontal cortex function among those aged 20–40 (Supplementary Figure 7). To standardize lipid values, the Yeo-Johnson transformation was trained on data from individuals within this age range and then applied to all other samples to ensure comparability [84, 86]. Following transformation, lipid values were binarized by assigning a value of 1 if they deviated beyond two standard deviations from the normalized reference distribution, and 0 otherwise.

To evaluate lipidomic variability across the lifespan, we calculated group-based entropy within 10-year age bins. For each age group, the proportion of individuals exhibiting lipid deviations (0 or 1) was computed for each lipid feature, and Shannon entropy was calculated based on these proportions. Group-level entropy values were then compared across adjacent age bins (e.g., 20–30 vs. 30–40, 30–40 vs. 40–50) to assess age-related changes in lipid variability.

In addition, we calculated entropy for each individual to understand how much their lipid profile differs from what is typical for their age. To do this, we first looked at how often each lipid showed deviations in healthy people of the same age group. Then, for each individual, we gave more weight to lipid deviations that are rare in healthy people of the same age. Using these weighted values, we calculated entropy to capture how unusual or variable each person’s lipid profile is, compared to normal patterns for their age. This allowed us to compare individuals with neurological disorders to healthy controls, to see if their lipid profiles show greater differences from what is normally expected with aging.

### Implementation details

The analysis pipeline was implemented in Python (v3.9.7, (59)). Numpy (v1.25.2, [92]), Pandas (v2.1.1, [93]) and Scipy (v1.11.3, [94]) were used for computational purposes and scikit-learn (v1.5.2, [86]) was used for the development of machine learning models. For visualization matplotlib (v3.8.0, [95]) and seaborn (v0.13.2, [96]) were used. For interpretation of models the SHAP library (v0.43.0, [90]) was used.

Code to reproduce the results are made available at https://github.com/ddlatumalea/DoliClock-2.0.

## Supplementary Materials

Supplementary Figures

Supplementary Table 1

Supplementary Table 2

Supplementary Table 3

Supplementary Tables 4 and 5

## References

[1] Schneider EL, Guralnik JM. The aging of America. Impact on health care costs. JAMA. 1990; 263:2335–40. 2109105

[2] Feigin VL, Vos T, Nichols E, Owolabi MO, Carroll WM, Dichgans M, Deuschl G, Parmar P, Brainin M, Murray C. The global burden of neurological disorders: translating evidence into policy. Lancet Neurol. 2020; 19:255–65. 10.1016/S1474-4422(19)30411-9 31813850 PMC9945815

[3] Centers for Disease Control and Prevention (CDC). Trends in aging--United States and worldwide. MMWR Morb Mortal Wkly Rep. 2003; 52:101–4. 12645839

[4] Hou Y, Dan X, Babbar M, Wei Y, Hasselbalch SG, Croteau DL, Bohr VA. Ageing as a risk factor for neurodegenerative disease. Nat Rev Neurol. 2019; 15:565–81. 10.1038/s41582-019-0244-7 31501588

[5] Troen BR. The biology of aging. Mt Sinai J Med. 2003; 70:3–22. 12516005

[6] Nguyen QD, Moodie EM, Forget MF, Desmarais P, Keezer MR, Wolfson C. Health Heterogeneity in Older Adults: Exploration in the Canadian Longitudinal Study on Aging. J Am Geriatr Soc. 2021; 69:678–87. 10.1111/jgs.16919 33155270

[7] Balcombe NR, Sinclair A. Ageing: definitions, mechanisms and the magnitude of the problem. Best Pract Res Clin Gastroenterol. 2001; 15:835–49. 10.1053/bega.2001.0244 11866480

[8] López-Otín C, Blasco MA, Partridge L, Serrano M, Kroemer G. Hallmarks of aging: An expanding universe. Cell. 2023; 186:243–78. 10.1016/j.cell.2022.11.001 36599349

[9] Sargent JR, Tocher DR, Bell JG. 4 - The Lipids. In: Halver JE, Hardy RW, editors. Fish Nutrition (Third Edition). San Diego: Academic Press; 2003; 181–257. https://www.sciencedirect.com/science/article/pii/B9780123196521500057.

[10] Sunshine H, Iruela-Arispe ML. Membrane lipids and cell signaling. Curr Opin Lipidol. 2017; 28:408–13. 10.1097/MOL.0000000000000443 28692598 PMC5776726

[11] Yu Q, He Z, Zubkov D, Huang S, Kurochkin I, Yang X, Halene T, Willmitzer L, Giavalisco P, Akbarian S, Khaitovich P. Lipidome alterations in human prefrontal cortex during development, aging, and cognitive disorders. Mol Psychiatry. 2020; 25:2952–69. 10.1038/s41380-018-0200-8 30089790 PMC7577858

[12] Jové M, Mota-Martorell N, Torres P, Portero-Otin M, Ferrer I, Pamplona R. New insights into human prefrontal cortex aging with a lipidomics approach. Expert Rev Proteomics. 2021; 18:333–44. 10.1080/14789450.2021.1940142 34098823

[13] McEwen BS, Morrison JH. The brain on stress: vulnerability and plasticity of the prefrontal cortex over the life course. Neuron. 2013; 79:16–29. 10.1016/j.neuron.2013.06.028 23849196 PMC3753223

[14] Kao YC, Ho PC, Tu YK, Jou IM, Tsai KJ. Lipids and Alzheimer's Disease. Int J Mol Sci. 2020; 21:1505. 10.3390/ijms21041505 32098382 PMC7073164

[15] Shichiri M. The role of lipid peroxidation in neurological disorders. J Clin Biochem Nutr. 2014; 54:151–60. 10.3164/jcbn.14-10 24895477 PMC4042144

[16] Read J, Perry BD, Moskowitz A, Connolly J. The contribution of early traumatic events to schizophrenia in some patients: a traumagenic neurodevelopmental model. Psychiatry. 2001; 64:319–45. 10.1521/psyc.64.4.319.18602 11822210

[17] Selemon LD, Zecevic N. Schizophrenia: a tale of two critical periods for prefrontal cortical development. Transl Psychiatry. 2015; 5:e623. 10.1038/tp.2015.115 26285133 PMC4564568

[18] Wong CT, Wais J, Crawford DA. Prenatal exposure to common environmental factors affects brain lipids and increases risk of developing autism spectrum disorders. Eur J Neurosci. 2015; 42:2742–60. 10.1111/ejn.13028 26215319

[19] Bittles AH, Glasson EJ. Clinical, social, and ethical implications of changing life expectancy in Down syndrome. Dev Med Child Neurol. 2004; 46:282–6. 10.1017/s0012162204000441 15077706

[20] Perkins EA, Berkman KA. Into the unknown: aging with autism spectrum disorders. Am J Intellect Dev Disabil. 2012; 117:478–96. 10.1352/1944-7558-117.6.478 23167487

[21] Hjorthøj C, Stürup AE, McGrath JJ, Nordentoft M. Years of potential life lost and life expectancy in schizophrenia: a systematic review and meta-analysis. Lancet Psychiatry. 2017; 4:295–301. 10.1016/S2215-0366(17)30078-0 28237639

[22] Hannum G, Guinney J, Zhao L, Zhang L, Hughes G, Sadda S, Klotzle B, Bibikova M, Fan JB, Gao Y, Deconde R, Chen M, Rajapakse I, et al. Genome-wide methylation profiles reveal quantitative views of human aging rates. Mol Cell. 2013; 49:359–67. 10.1016/j.molcel.2012.10.016 23177740 PMC3780611

[23] Horvath S. DNA methylation age of human tissues and cell types. Genome Biol. 2013; 14:R115. 10.1186/gb-2013-14-10-r115 24138928 PMC4015143

[24] Horvath S, Raj K. DNA methylation-based biomarkers and the epigenetic clock theory of ageing. Nat Rev Genet. 2018; 19:371–84. 10.1038/s41576-018-0004-3 29643443

[25] Meyer DH, Schumacher B. BiT age: A transcriptome-based aging clock near the theoretical limit of accuracy. Aging Cell. 2021; 20:e13320. 10.1111/acel.13320 33656257 PMC7963339

[26] Johnson AA, Shokhirev MN, Lehallier B. The protein inputs of an ultra-predictive aging clock represent viable anti-aging drug targets. Ageing Res Rev. 2021; 70:101404. 10.1016/j.arr.2021.101404 34242807

[27] Unfried M, Ng LF, Cazenave-Gassiot A, Batchu KC, Kennedy BK, Wenk MR, Tolwinski N, Gruber J. LipidClock: A Lipid-Based Predictor of Biological Age. Front Aging. 2022; 3:828239. 10.3389/fragi.2022.828239 35821819 PMC9261347

[28] Hwangbo N, Zhang X, Raftery D, Gu H, Hu SC, Montine TJ, Quinn JF, Chung KA, Hiller AL, Wang D, Fei Q, Bettcher L, Zabetian CP, et al. A Metabolomic Aging Clock Using Human Cerebrospinal Fluid. J Gerontol A Biol Sci Med Sci. 2022; 77:744–54. 10.1093/gerona/glab212 34382643 PMC8974344

[29] Urban A, Sidorenko D, Zagirova D, Kozlova E, Kalashnikov A, Pushkov S, Naumov V, Sarkisova V, Leung GHD, Leung HW, Pun FW, Ozerov IV, Aliper A, et al. Precious1GPT: multimodal transformer-based transfer learning for aging clock development and feature importance analysis for aging and age-related disease target discovery. Aging (Albany NY). 2023; 15:4649–66. 10.18632/aging.204788 37315204 PMC10292881

[30] Levine ME, Lu AT, Quach A, Chen BH, Assimes TL, Bandinelli S, Hou L, Baccarelli AA, Stewart JD, Li Y, Whitsel EA, Wilson JG, Reiner AP, et al. An epigenetic biomarker of aging for lifespan and healthspan. Aging (Albany NY). 2018; 10:573–91. 10.18632/aging.101414 29676998 PMC5940111

[31] Lu AT, Quach A, Wilson JG, Reiner AP, Aviv A, Raj K, Hou L, Baccarelli AA, Li Y, Stewart JD, Whitsel EA, Assimes TL, Ferrucci L, Horvath S. DNA methylation GrimAge strongly predicts lifespan and healthspan. Aging (Albany NY). 2019; 11:303–27. 10.18632/aging.101684 30669119 PMC6366976

[32] Horvath S, Garagnani P, Bacalini MG, Pirazzini C, Salvioli S, Gentilini D, Di Blasio AM, Giuliani C, Tung S, Vinters HV, Franceschi C. Accelerated epigenetic aging in Down syndrome. Aging Cell. 2015; 14:491–5. 10.1111/acel.12325 25678027 PMC4406678

[33] Cole JH, Annus T, Wilson LR, Remtulla R, Hong YT, Fryer TD, Acosta-Cabronero J, Cardenas-Blanco A, Smith R, Menon DK, Zaman SH, Nestor PJ, Holland AJ. Brain-predicted age in Down syndrome is associated with beta amyloid deposition and cognitive decline. Neurobiol Aging. 2017; 56:41–9. 10.1016/j.neurobiolaging.2017.04.006 28482213 PMC5476346

[34] Kirkpatrick B, Kennedy BK. Accelerated aging in schizophrenia and related disorders: Future research. Schizophr Res. 2018; 196:4–8. 10.1016/j.schres.2017.06.034 28689755

[35] Higgins-Chen AT, Boks MP, Vinkers CH, Kahn RS, Levine ME. Schizophrenia and Epigenetic Aging Biomarkers: Increased Mortality, Reduced Cancer Risk, and Unique Clozapine Effects. Biol Psychiatry. 2020; 88:224–35. 10.1016/j.biopsych.2020.01.025 32199607 PMC7368835

[36] Teeuw J, Ori APS, Brouwer RM, de Zwarte SMC, Schnack HG, Hulshoff Pol HE, Ophoff RA. Accelerated aging in the brain, epigenetic aging in blood, and polygenic risk for schizophrenia. Schizophr Res. 2021; 231:189–97. 10.1016/j.schres.2021.04.005 33882370

[37] Okazaki S, Otsuka I, Numata S, Horai T, Mouri K, Boku S, Ohmori T, Sora I, Hishimoto A. Epigenetic clock analysis of blood samples from Japanese schizophrenia patients. NPJ Schizophr. 2019; 5:4. 10.1038/s41537-019-0072-1 30814520 PMC6393510

[38] Happé F, Charlton RA. Aging in autism spectrum disorders: a mini-review. Gerontology. 2012; 58:70–8. 10.1159/000329720 21865667

[39] Thrush KL, Bennett DA, Gaiteri C, Horvath S, Dyck CHV, Higgins-Chen AT, Levine ME. Aging the brain: multi-region methylation principal component based clock in the context of Alzheimer's disease. Aging (Albany NY). 2022; 14:5641–68. 10.18632/aging.204196 35907208 PMC9365556

[40] Levine ME, Lu AT, Bennett DA, Horvath S. Epigenetic age of the pre-frontal cortex is associated with neuritic plaques, amyloid load, and Alzheimer's disease related cognitive functioning. Aging (Albany NY). 2015; 7:1198–211. 10.18632/aging.100864 26684672 PMC4712342

[41] Ren X, Kuan PF. RNAAgeCalc: A multi-tissue transcriptional age calculator. PLoS One. 2020; 15:e0237006. 10.1371/journal.pone.0237006 32750074 PMC7402472

[42] Martínez-Magaña JJ, Krystal JH, Girgenti MJ, Núnez-Ríos DL, Nagamatsu ST, Andrade-Brito DE, Group TSB, Montalvo-Ortiz JL, and Traumatic Stress Brain Research Group. Decoding the role of transcriptomic clocks in the human prefrontal cortex. medRxiv. 2023. 10.1101/2023.04.19.23288765 37163025 PMC10168432

[43] O'Brien JS, Sampson EL. Lipid composition of the normal human brain: gray matter, white matter, and myelin. J Lipid Res. 1965; 6:537–44. 5865382

[44] Naudí A, Cabré R, Jové M, Ayala V, Gonzalo H, Portero-Otín M, Ferrer I, Pamplona R. Lipidomics of human brain aging and Alzheimer's disease pathology. Int Rev Neurobiol. 2015; 122:133–89. 10.1016/bs.irn.2015.05.008 26358893

[45] Moqri M, Herzog C, Poganik JR, Ying K, Justice JN, Belsky DW, Higgins-Chen AT, Chen BH, Cohen AA, Fuellen G, Hägg S, Marioni RE, Widschwendter M, et al. Validation of biomarkers of aging. Nat Med. 2024; 30:360–72. 10.1038/s41591-023-02784-9 38355974 PMC11090477

[46] Moqri M, Herzog C, Poganik JR, Justice J, Belsky DW, Higgins-Chen A, Moskalev A, Fuellen G, Cohen AA, Bautmans I, Widschwendter M, Ding J, Fleming A, et al, and Biomarkers of Aging Consortium. Biomarkers of aging for the identification and evaluation of longevity interventions. Cell. 2023; 186:3758–75. 10.1016/j.cell.2023.08.003 37657418 PMC11088934

[47] Higgins-Chen AT, Thrush KL, Wang Y, Minteer CJ, Kuo PL, Wang M, Niimi P, Sturm G, Lin J, Moore AZ, Bandinelli S, Vinkers CH, Vermetten E, et al. A computational solution for bolstering reliability of epigenetic clocks: Implications for clinical trials and longitudinal tracking. Nat Aging. 2022; 2:644–61. 10.1038/s43587-022-00248-2 36277076 PMC9586209

[48] Mason D, Ronald A, Ambler A, Caspi A, Houts R, Poulton R, Ramrakha S, Wertz J, Moffitt TE, Happé F. Autistic traits are associated with faster pace of aging: Evidence from the Dunedin study at age 45. Autism Res. 2021; 14:1684–94. 10.1002/aur.2534 34042279 PMC8328948

[49] Chojnacki T, Dallner G. The biological role of dolichol. Biochem J. 1988; 251:1–9. 10.1042/bj2510001 3291859 PMC1148956

[50] Welti M. Regulation of dolichol-linked glycosylation. Glycoconj J. 2013; 30:51–6. 10.1007/s10719-012-9417-y 22717794

[51] Goldstein JL, Brown MS. Regulation of the mevalonate pathway. Nature. 1990; 343:425–30. 10.1038/343425a0 1967820

[52] Sakakihara Y, Volpe JJ. Dolichol deposition in developing mammalian brain: content of free and fatty-acylated dolichol and proportion of specific isoprenologues. Brain Res. 1984; 316:255–62. 10.1016/0165-3806(84)90310-9 6467016

[53] Sakakihara Y, Imabayashi T, Suzuki Y, Kamoshita S. Elevated levels of dolichol in the brains of mucopolysaccharidosis and related disorders. Mol Chem Neuropathol. 1994; 22:97–103. 10.1007/BF03160098 7916772

[54] Brooksbank BW, Martinez M. Lipid abnormalities in the brain in adult Down's syndrome and Alzheimer's disease. Mol Chem Neuropathol. 1989; 11:157–85. 10.1007/BF03160049 2534986

[55] Kurup RK, Kurup PA. A hypothalamic digoxin-mediated model for autism. Int J Neurosci. 2003; 113:1537–59. 10.1080/00207450390231482 14585753

[56] Zhang Y, Appelkvist EL, Kristensson K, Dallner G. The lipid compositions of different regions of rat brain during development and aging. Neurobiol Aging. 1996; 17:869–75. 10.1016/s0197-4580(96)00076-0 9363798

[57] Hooff GP, Wood WG, Kim JH, Igbavboa U, Ong WY, Muller WE, Eckert GP. Brain isoprenoids farnesyl pyrophosphate and geranylgeranyl pyrophosphate are increased in aged mice. Mol Neurobiol. 2012; 46:179–85. 10.1007/s12035-012-8285-6 22692983

[58] Shen X, Wang C, Zhou X, Zhou W, Hornburg D, Wu S, Snyder MP. Nonlinear dynamics of multi-omics profiles during human aging. Nat Aging. 2024; 4:1619–34. 10.1038/s43587-024-00692-2 39143318 PMC11564093

[59] Meyer DH, Schumacher B. Aging clocks based on accumulating stochastic variation. Nat Aging. 2024; 4:871–85. 10.1038/s43587-024-00619-x 38724736 PMC11186771

[60] Tarkhov AE, Denisov KA, Fedichev PO. Aging clocks, entropy, and the limits of age-reversal. bioRxiv. 2022; 2022.02.06.479300. https://www.biorxiv.org/content/10.1101/2022.02.06.479300v2.

[61] Tarkhov AE, Lindstrom-Vautrin T, Zhang S, Ying K, Moqri M, Zhang B, Tyshkovskiy A, Levy O, Gladyshev VN. Nature of epigenetic aging from a single-cell perspective. Nat Aging. 2024; 4:854–70. 10.1038/s43587-024-00616-0 38724733

[62] Crick DC, Rip JW. Age-associated changes in dolichol and dolichyl phosphate metabolism in the kidneys and liver of mice. Biochim Biophys Acta. 1989; 1004:180–6. 10.1016/0005-2760(89)90266-x 2752016

[63] Pullarkat RK, Reha H, Pullarkat PS. Age-associated increase of free dolichol levels in mice. Biochim Biophys Acta. 1984; 793:494–6. 10.1016/0005-2760(84)90269-8 6712985

[64] Wood WG, Sun GY, Schroeder F. Membrane properties of dolichol in different age groups of mice. Chem Phys Lipids. 1989; 51:219–26. 10.1016/0009-3084(89)90009-1 2611963

[65] Dini B, Dolfi C, Santucci V, Cavallini G, Donati A, Gori Z, Maccheroni M, Bergamini E. Effects of ageing and increased haemolysis on the levels of dolichol in rat spleen. Exp Gerontol. 2001; 37:99–105. 10.1016/s0531-5565(01)00156-5 11738151

[66] Marino M, Pallottini V, D'Eramo C, Cavallini G, Bergamini E, Trentalance A. Age-related changes of cholesterol and dolichol biosynthesis in rat liver. Mech Ageing Dev. 2002; 123:1183–9. 10.1016/s0047-6374(02)00009-x 12044967

[67] Dolfi C, Bergamini E, Carresi C, Cavallini G, Donati A, Maccheroni M, Parentini I, Marino M, Gori Z. The age-related accumulation of dolichol in rat liver may correlate with expectation of life. Biogerontology. 2003; 4:113–8. 10.1023/a:1023304021679 12766536

[68] Cavallini G, Sgarbossa A, Parentini I, Bizzarri R, Donati A, Lenci F, Bergamini E. Dolichol: A Component of the Cellular Antioxidant Machinery. Lipids. 2016; 51:477–86. 10.1007/s11745-016-4137-x 26968401

[69] Ishinaga M. Effects of aging and diet on the accumulation of dolichol in rat tissues. Biochem Cell Biol. 1996; 74:265–70. 10.1139/o96-028 9213436

[70] Keller RK, Nellis SW. Quantitation of dolichyl phosphate and dolichol in major organs of the rat as a function of age. Lipids. 1986; 21:353–5. 10.1007/BF02535700 3724371

[71] Sternick SM, Massie HR, Whitney SJ. Changes with ageing in total dolichol and dolichol fractions in Drosophila. Mech Ageing Dev. 1993; 67:91–9. 10.1016/0047-6374(93)90114-7 8469036

[72] Carroll KK, Guthrie N, Ravi K. Dolichol: function, metabolism, and accumulation in human tissues. Biochem Cell Biol. 1992; 70:382–4. 10.1139/o92-059 1449704

[73] Edlund C, Söderberg M, Kristensson K, Dallner G. Ubiquinone, dolichol, and cholesterol metabolism in aging and Alzheimer's disease. Biochem Cell Biol. 1992; 70:422–8. 10.1139/o92-065 1449707

[74] Andersson M, Appelkvist EL, Kristensson K, Dallner G. Distribution of dolichol and dolichyl phosphate in human brain. J Neurochem. 1987; 49:685–91. 10.1111/j.1471-4159.1987.tb00948.x 3612118

[75] Sakakihara Y, Volpe JJ. Dolichol in human brain: regional and developmental aspects. J Neurochem. 1985; 44:1535–40. 10.1111/j.1471-4159.1985.tb08792.x 3989548

[76] Zhang H, Ohyama K, Boudet J, Chen Z, Yang J, Zhang M, Muranaka T, Maurel C, Zhu JK, Gong Z. Dolichol biosynthesis and its effects on the unfolded protein response and abiotic stress resistance in Arabidopsis. Plant Cell. 2008; 20:1879–98. 10.1105/tpc.108.061150 18612099 PMC2518237

[77] Parentini I, Cavallini G, Donati A, Gori Z, Bergamini E. Accumulation of dolichol in older tissues satisfies the proposed criteria to be qualified a biomarker of aging. J Gerontol A Biol Sci Med Sci. 2005; 60:39–43. 10.1093/gerona/60.1.39 15741281

[78] Marino M, Dolfi C, Paradiso C, Cavallini G, Masini M, Gori Z, Pollera M, Trentalance A, Bergamini E. Age-dependent accumulation of dolichol in rat liver: is tissue dolichol a biomarker of aging? J Gerontol A Biol Sci Med Sci. 1998; 53:B87–93. 10.1093/gerona/53a.2.b87 9520903

[79] Krištić J, Vučković F, Menni C, Klarić L, Keser T, Beceheli I, Pučić-Baković M, Novokmet M, Mangino M, Thaqi K, Rudan P, Novokmet N, Sarac J, et al. Glycans are a novel biomarker of chronological and biological ages. J Gerontol A Biol Sci Med Sci. 2014; 69:779–89. 10.1093/gerona/glt190 24325898 PMC4049143

[80] Cantagrel V, Lefeber DJ. From glycosylation disorders to dolichol biosynthesis defects: a new class of metabolic diseases. J Inherit Metab Dis. 2011; 34:859–67. 10.1007/s10545-011-9301-0 21384228 PMC3137772

[81] Bergamini E, Bizzarri R, Cavallini G, Cerbai B, Chiellini E, Donati A, Gori Z, Manfrini A, Parentini I, Signori F, Tamburini I. Ageing and oxidative stress: a role for dolichol in the antioxidant machinery of cell membranes? J Alzheimers Dis. 2004; 6:129–35. 10.3233/jad-2004-6204 15096696

[82] Rip JW, Blais MM, Jiang LW. Low-density lipoprotein as a transporter of dolichol intermediates in the mammalian circulation. Biochem J. 1994; 297:321–5. 10.1042/bj2970321 8297338 PMC1137832

[83] Mantel-Teeuwisse AK, Kloosterman JM, Maitland-van der Zee AH, Klungel OH, Porsius AJ, de Boer A. Drug-Induced lipid changes: a review of the unintended effects of some commonly used drugs on serum lipid levels. Drug Saf. 2001; 24:443–56. 10.2165/00002018-200124060-00003 11368251

[84] Yeo IK, Johnson RA. A New Family of Power Transformations to Improve Normality or Symmetry. Biometrika. Oxford University Press, Biometrika Trust. 2000; 87:954–9. https://www.jstor.org/stable/2673623.

[85] Jolliffe IT. Principal Component Analysis. New York, NY: Springer; 1986. http://link.springer.com/10.1007/978-1-4757-1904-8.

[86] Pedregosa F, Varoquaux G, Gramfort A, Michel V, Thirion B, Grisel O, Blondel M, Prettenhofer P, Weiss R, Dubourg V, Vanderplas J, Passos A, Cournapeau D, et al. Scikit-learn: Machine Learning in Python. J Mach Learn Res. 2011; 12:2825–30.

[87] Taleb NN, Zalloua P, Elbassioni K, Henschel A, Platt DE. Informational Rescaling of PCA Maps with Application to Genetic Distance. arXiv. 2024. http://arxiv.org/abs/2303.12654. 10.1016/j.csbj.2024.11.042 PMC11719279 39802212

[88] Platt DE, Artinian H, Mouzaya F, Khalil W, Kamar FG, Matisoo-Smith E, Calafell F, Taleb NN, Zalloua P. Autosomal genetics and Y-chromosome haplogroup L1b-M317 reveal Mount Lebanon Maronites as a persistently non-emigrating population. Eur J Hum Genet. 2021; 29:581–92. 10.1038/s41431-020-00765-x 33273712 PMC8182888

[89] Zou H, Hastie T. Regularization and Variable Selection Via the Elastic Net. J R Stat Soc Ser B Stat Methodol. 2005; 67:301–20. 10.1111/j.1467-9868.2005.00503.x

[90] Lundberg SM, Lee SI. A unified approach to interpreting model predictions. Proceedings of the 31st International Conference on Neural Information Processing Systems. Red Hook, NY, USA: Curran Associates Inc. 2017; 4768–77. 10.48550/arXiv.1705.07874

[91] Shannon CE. A mathematical theory of communication. Bell Syst Tech J. 1948; 27:379–423.

[92] Harris CR, Millman KJ, van der Walt SJ, Gommers R, Virtanen P, Cournapeau D, Wieser E, Taylor J, Berg S, Smith NJ, Kern R, Picus M, Hoyer S, et al. Array programming with NumPy. Nature. 2020; 585:357–62. 10.1038/s41586-020-2649-2 32939066 PMC7759461

[93] Team T pandas development. pandas-dev/pandas: Pandas. Zenodo. 2024. https://zenodo.org/records/13819579.

[94] McKinney W. Data Structures for Statistical Computing in Python. scipy. 2010. https://proceedings.scipy.org/articles/Majora-92bf1922-00a.

[95] Hunter JD. Matplotlib: A 2D Graphics Environment. Comput Sci Eng. 2007; 9:90–5. 10.1109/MCSE.2007.55

[96] Waskom ML. seaborn: statistical data visualization. J Open Source Softw. 2021; 6:3021. 10.21105/joss.03021

