# Supplementary material for: DoliClock: a lipid-based aging clock reveals accelerated aging in neurological disorders
Source: Aging (Albany NY). 2025 Jun 4;17(6):1405–28. doi: 10.18632/aging.206266 (PMC12245196; doi:10.18632/aging.206266)
Supplement: Supplementary Figures [file aging-17-206266-s001.pdf]

SUPPLEMENTARY FIGURES

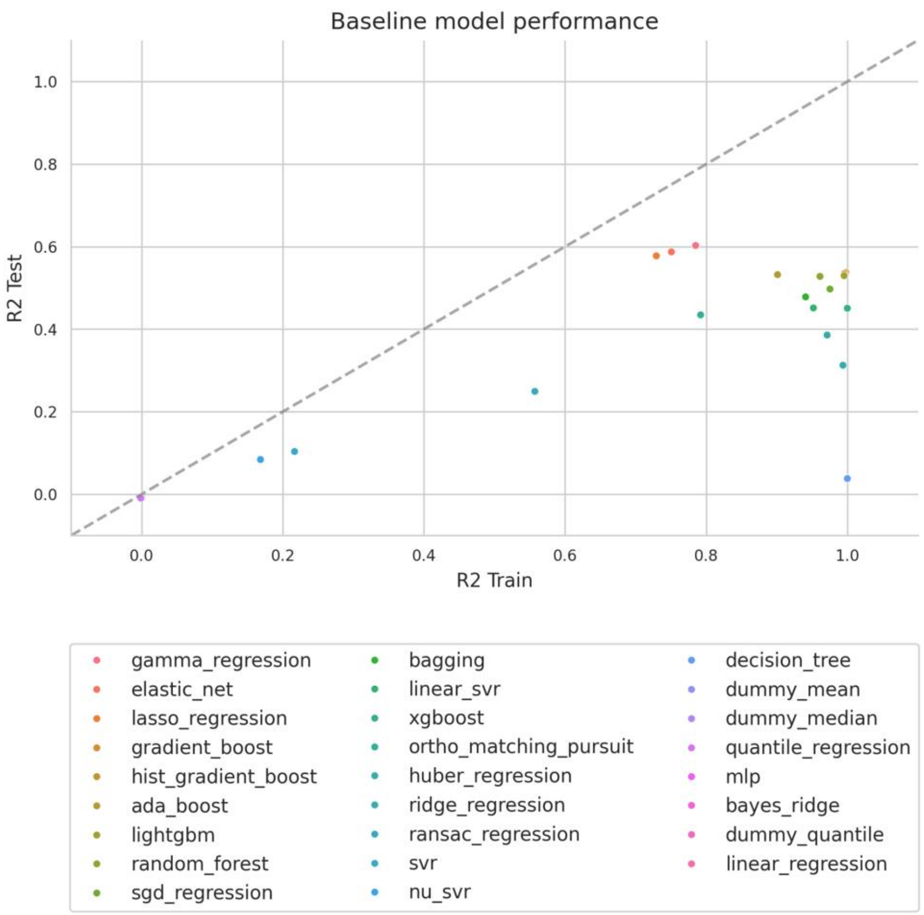

Supplementary Figure 1. Comparison of 26 models based on their R^2 test score versus training score. Each point represents a model, illustrating its performance on both the training and test datasets.

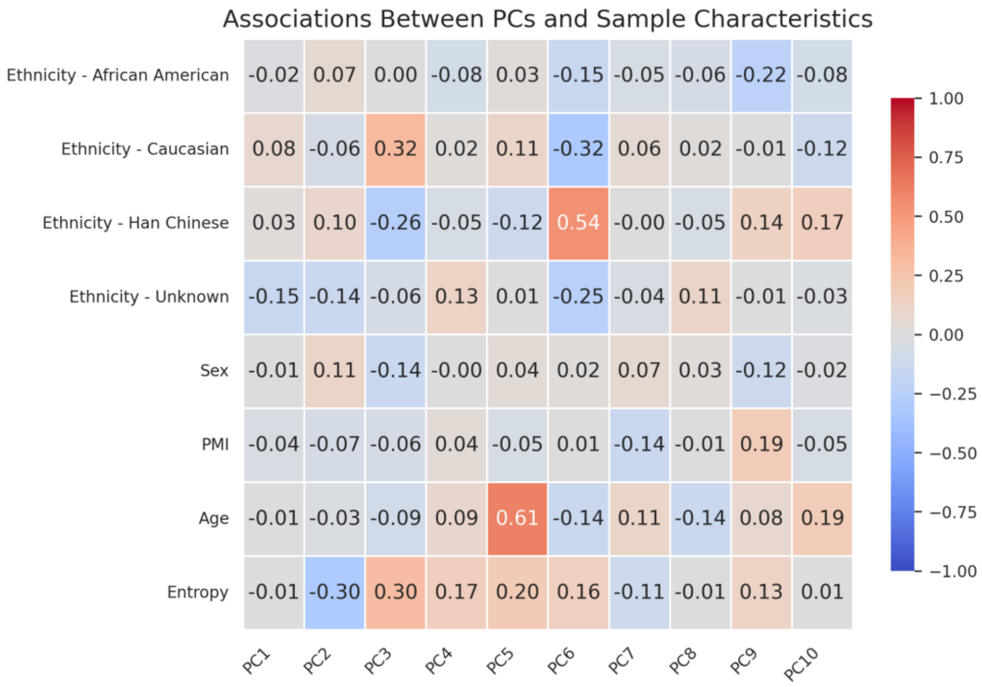

Supplementary Figure 2. Correlation heatmap of principal components and metadata from the individuals. The Pearson correlation coefficient was used to calculate correlation. Warmer colors indicate positive correlations, whereas cooler colors indicate negative correlations.

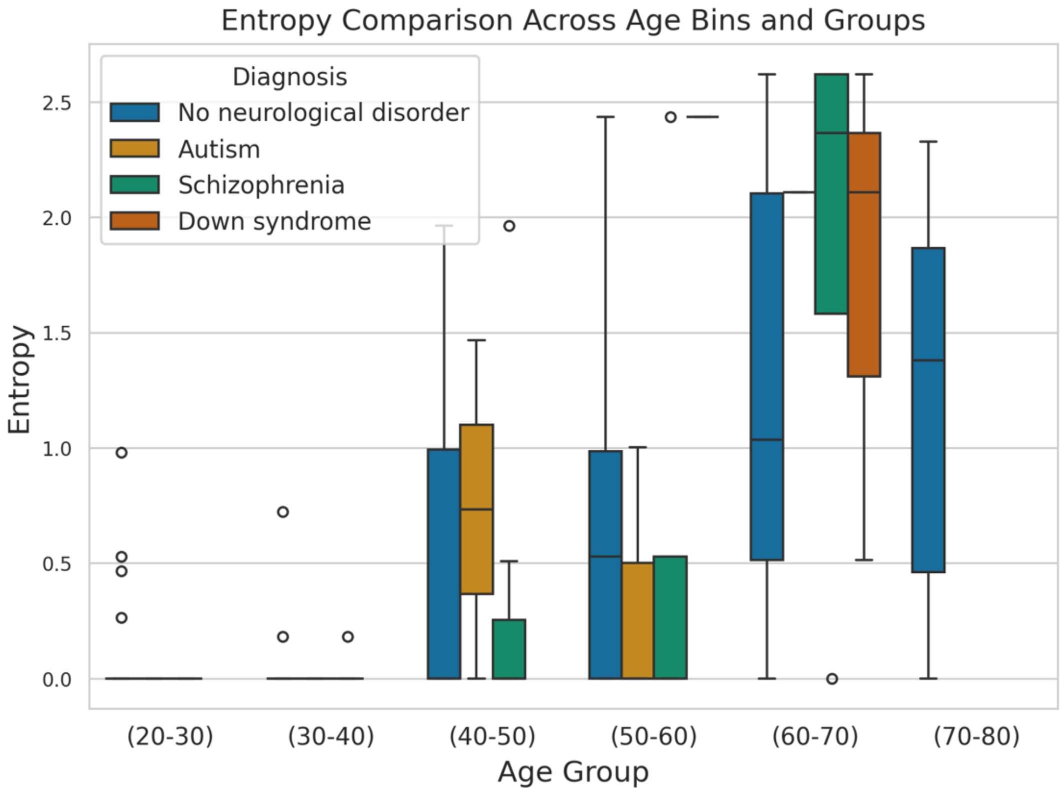

Supplementary Figure 3. Sample-wise entropy across neurological conditions and age groups. Boxplots of sample-wise entropy values for samples without neurological disorder, and for samples with autism spectrum disorder, schizophrenia, and Down syndrome stratified by age (only significant *P*-values are annotated: $P < 0.05 = ^{*}$, $P < 0.01 = ^{**}$, $P < 0.001 = ^{***}$).

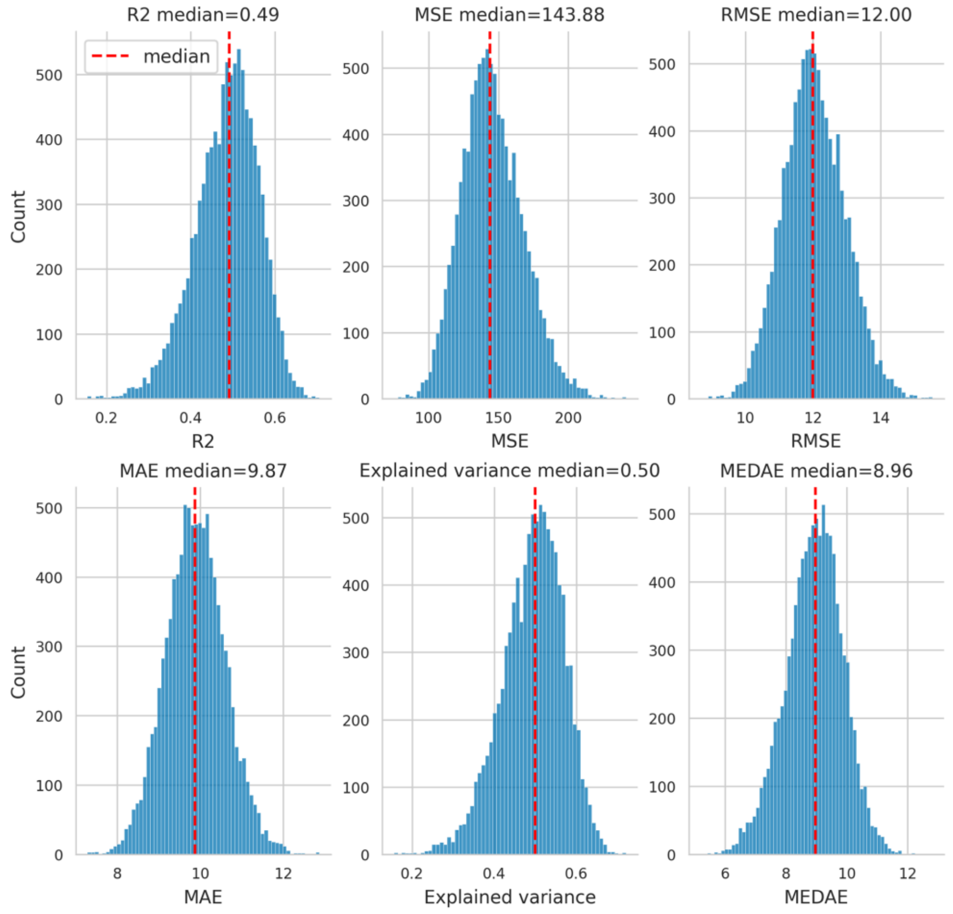

Supplementary Figure 4. Distribution of all model scores, with the median value indicated.

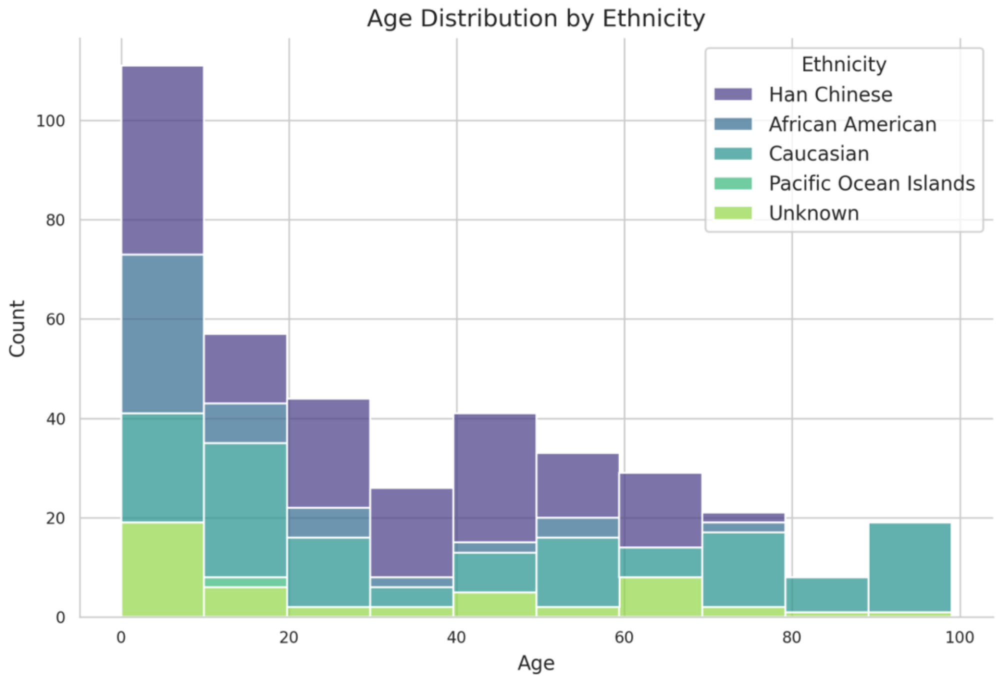

Supplementary Figure 5. Histogram showing the age distribution across different ethnicities. Age is divided into bins of 10 years, and each ethnicity is represented by a distinct color to highlight demographic variation within the dataset.

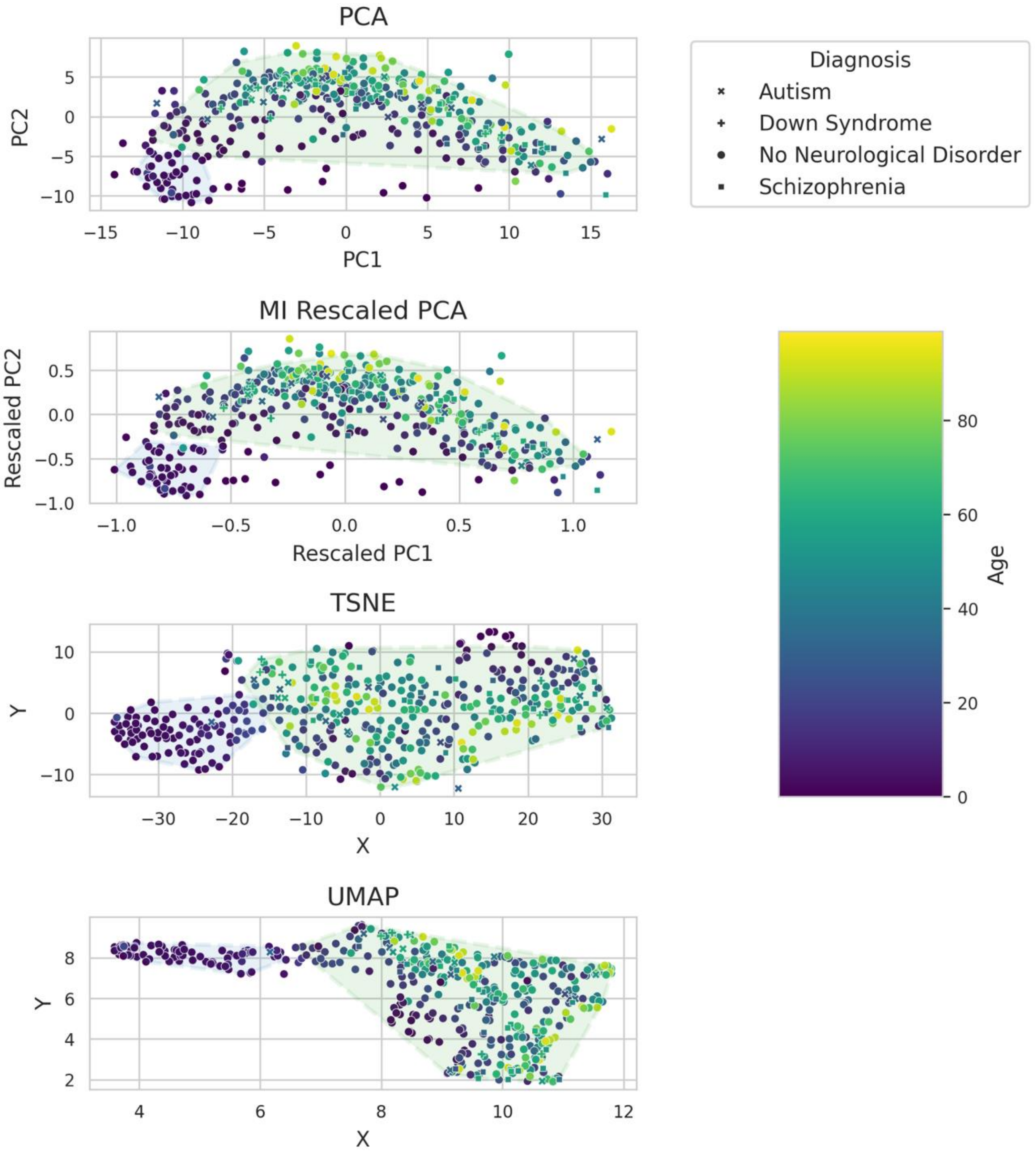

Supplementary Figure 6. Scatter plots illustrating dimensionality reduction techniques applied to the dataset. Clusters were identified using DBScan and visualized with convex hulls. The figure presents PCA, PCA with Mutual Information (MI) rescaling, t-SNE, and UMAP, each displaying sample clustering based on all lipid species. The axes represent the first two dimensions of each respective method.

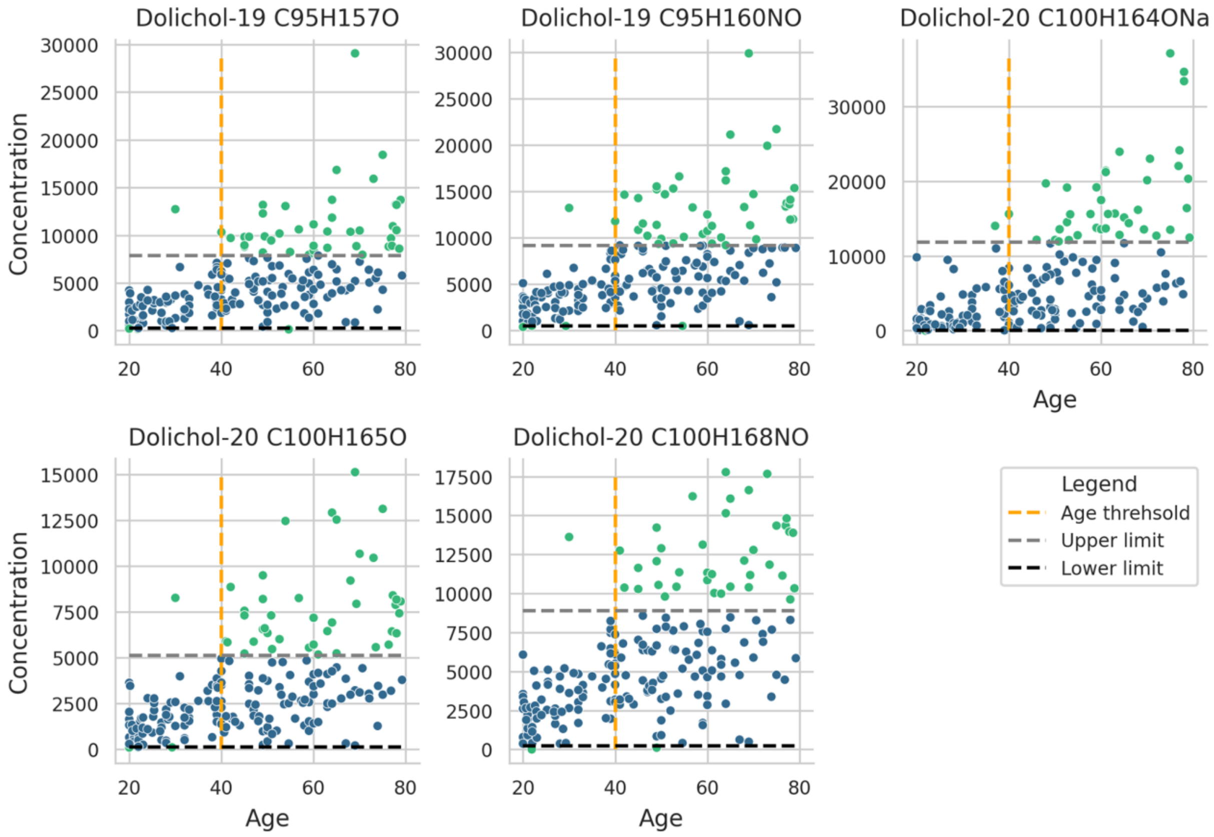

Supplementary Figure 7. Age-related variability in dolichol distributions. Each plot visualizes lipid distributions relative to a reference group (ages 20–40), standardized using Yeo-Johnson transformation. Individuals marked in blue have lipid profiles within two standard deviations of the reference distribution, while individuals marked in green deviate beyond this threshold.
