# Supplementary material for: DoliClock: a lipid-based aging clock reveals accelerated aging in neurological disorders
Source: Aging (Albany NY). 2025 Jun 4;17(6):1405–28. doi: 10.18632/aging.206266 (PMC12245196; doi:10.18632/aging.206266)
Supplement: Supplementary Tables 4 and 5 [file aging-17-206266-s005.pdf]

SUPPLEMENTARY TABLES

Supplementary Table 4. Log fold changes of dolichol concentration across different groups.

lipid_index	LogFC [0–20] [20–40]	LogFC [20–40] [40–60]	LogFC [40–60] [60–80]	LogFC [60–80] [80–100]
Dolichol-19_C95H157O	1.082794112	1.02362787	0.579947149	0.369038402
Dolichol-19_C95H160NO	1.484802507	1.001635751	0.485293785	0.318561547
Dolichol-20_C100H164ONa	1.006039152	1.292070672	0.937477928	0.411000008
Dolichol-20_C100H165O	1.532039971	0.997398856	0.583720461	0.412647358
Dolichol-20_C100H168NO	1.509776453	0.819964455	0.55020163	0.270673998

Supplementary Table 5. Demographics of the original dataset, including sample counts by neurological disorder status (No Neurological Disorder, Down Syndrome, Autism, Schizophrenia).

	No neurological disorder	Down syndrome	Autism	Schizophrenia
Count	403	5	17	27
**Gender**				
Male	260	3	12	20
Female	141	2	5	7
Unknown	2	-	–	–
**Age**				
<0	12	–	–	–
0–20	169	–	2	–
20–30	45	–	3	4
30–40	26	–	6	5
40–50	41	–	2	7
50–60	33	2	3	7
60–70	30	3	1	4
70–80	21	–	–	–
80+	26	–	–	–
**Ethnicity**				
Han Chinese	148	–	–	–
Caucasian	136	5	–	1
African American	61	–	–	–
Hispanic	3	–	–	–
Pacific Ocean Islands	2	–	–	–
Unknown	53	–	17	26
**Other**				
PMI median (std)	14 (5.14)	6.167 (2.12)	21.08 (8.42)	19.5 (10.68)

Gender, age groups, ethnicity, and postmortem interval (PMI; median ± SD assumed to be in hours) are detailed. Dashes ("–") indicate unavailable data.
